# Reducing the energy cost of walking in older adults using a passive hip flexion device

**DOI:** 10.1186/s12984-019-0599-4

**Published:** 2019-10-15

**Authors:** Fausto A. Panizzolo, Chiara Bolgiani, Laura Di Liddo, Eugenio Annese, Giuseppe Marcolin

**Affiliations:** 1 Moveo Walks, 12 Remington Street, Cambridge, MA 02138 USA; 2 Department of Biomedical Sciences, University of Padova, Via Marzolo 3, 35131 Padua, Italy

**Keywords:** Older adults, Exoskeletons, Walking, Metabolic power

## Abstract

**Background:**

Elevated energy cost is a hallmark feature of gait in older adults. As such, older adults display a general avoidance of walking which contributes to declining health status and risk of morbidity. Exoskeletons offer a great potential for lowering the energy cost of walking, however their complexity and cost often limit their use. To overcome some of these issues, in the present work we propose a passive wearable assistive device, namely Exoband, that applies a torque to the hip flexors thus reducing the net metabolic power of wearers.

**Methods:**

Nine participants (age: 62.1 ± 5.6 yr; height: 1.71 ± 0.05 m; weight: 76.3 ± 11.9 kg) walked on a treadmill at a speed of 1.1 m/s with and without the Exoband. Metabolic power was measured by indirect calorimetry and spatio-temporal parameters measured using an optical measurement system. Heart rate and ratings of perceived exertion were recorded during data collection to monitor relative intensity of the walking trials.

**Results:**

The Exoband was able to provide a consistent torque (~ 0.03–0.05 Nm/kg of peak torque) to the wearers. When walking with the Exoband, participants displayed a lower net metabolic power with respect to free walking (− 3.3 ± 3.0%; *p* = 0.02). There were no differences in spatio-temporal parameters or relative intensities when walking with or without the Exoband.

**Conclusions:**

This study demonstrated that it is possible to reduce metabolic power during walking in older adults with the assistance of a passive device that applies a torque to the hip joint. Wearable, lightweight and low-cost devices such as the Exoband have the potential to make walking less metabolically demanding for older individuals.

## Background

The reduction in walking function experienced by older adults impacts quality of life, health status, and predicts life expectancy. As adults age, difficulty walking affects relative independence and the ability to execute daily tasks in an autonomous way [1], also representing a major burden to their health status and risk of morbidity. As a consequence, reduced walking in older adults is a major contributing factor to a variety of medical issues such as high blood pressure, obesity, and more severe conditions such as cardiovascular diseases and diabetes [2–4].

Elevated energy cost is a hallmark feature of gait in older adults and is most likely caused by multiple factors, including changes in neuromuscular and gait mechanics [5]. Elevated walking energy costs have also been shown to result in a general avoidance of walking and other activities in older adults [6]. Activity avoidance increases morbidity and mortality risk [7]. As such, lowering walking energy cost in older adults is predicted to significantly improve health, quality of life and life expectancy in older adults.

Recent engineering advancements coupled with musculoskeletal research have resulted in novel solutions to assist human walking. Exoskeletons offer great potential for lowering the energy cost of walking, reducing fatigue and mitigating mechanical stress on joints and bones [8–12]. Yet despite significant advancements in this field, exoskeletons that reduce metabolic cost of walking still present several shortcomings and are not widely adopted by consumers. Current devices are heavy, cumbersome to wear, and require trained personnel to be operated and maintained [13, 14]. They are also powered by large batteries that drain quickly and these, together with the electronic components needed to implement different control architectures, can be very costly. To overcome some of these limitations, recent studies have shown the potential of passive exoskeletons (devices that do not include motors and batteries) to assist walking [8] and running [15, 16]. These studies highlighted that, despite years of human evolution, it is possible to reduce the metabolic cost of gait by means of passive devices that store and release mechanical energy generated by the body during specific phases of the gait cycle.

Building on the foundation of this previous work, this manuscript presents a passive hip device composed of textile, thus making it extremely lightweight and easy to wear. The aim of this study is to determine whether a simple device that assists hip flexion can reduce the metabolic cost of walking in older adults. The choice to design a device helping hip flexion in the elderly was decided for two main reasons. The first is that it has been established previously that aging causes an increased reliance on the hip rather than on the ankle to power walking [17, 18]; the second is that a relevant simulation study on powered exoskeletons [19] indicated that assisting hip flexion provides greater metabolic savings with respect to other joints. Research in the exoskeleton field has also highlighted that physiological and neurological differences between individuals can cause divergent metabolic responses to the same device [20–22], and that responses can change considerably during the course of adaptation [23, 24], thus underlining the importance of an individualized level of assistance applied by the device [25, 26]. As such, we evaluated three different levels of assistance associated with a specific force index: LOW (0.3 N/kg), MED (0.5 N/kg) and HIGH (0.7 N/kg) applied by our device in a group of healthy community dwelling older adults.

## Materials and methods

### Exoband design

**Fig. 1:**
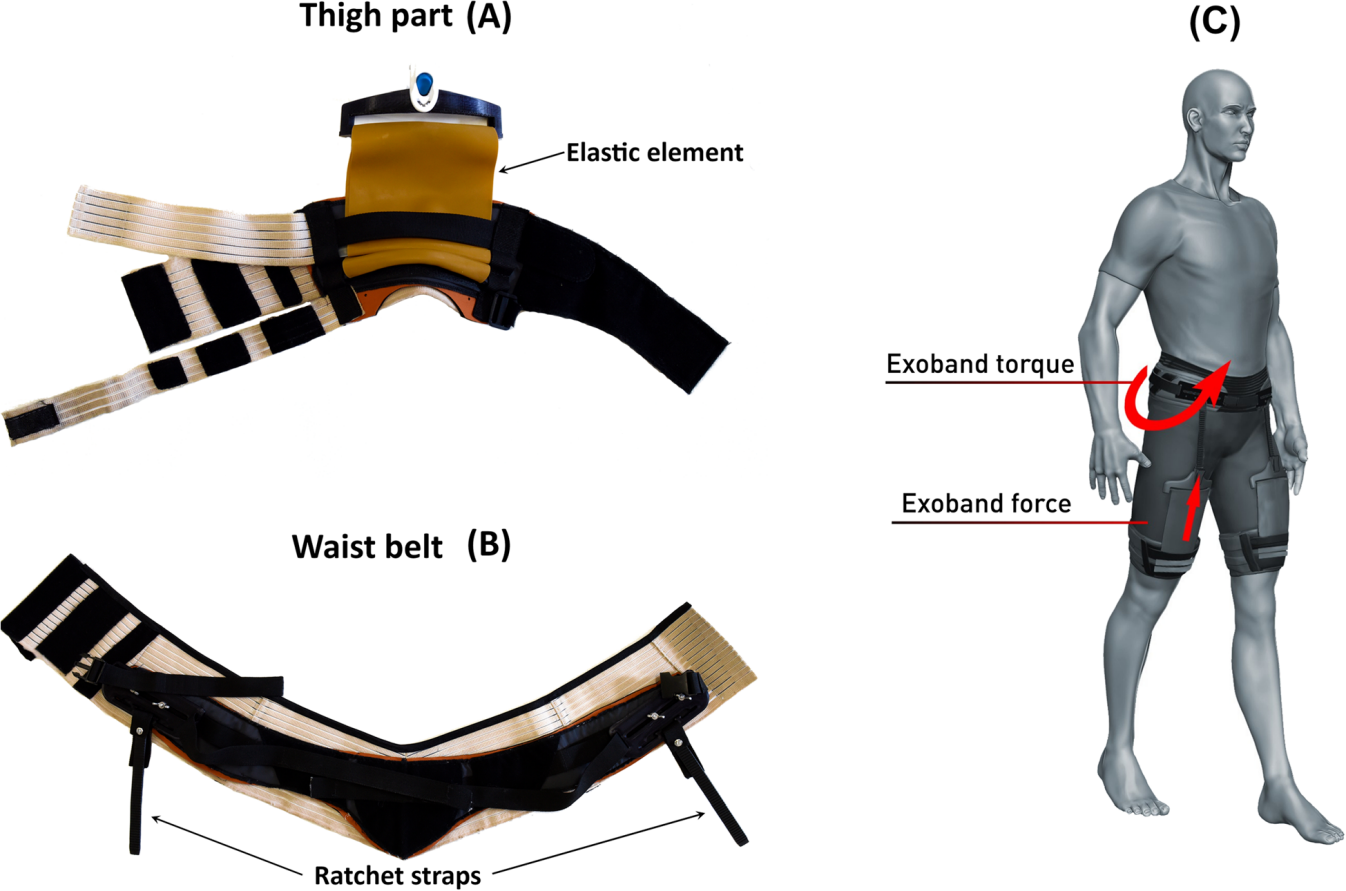
Exoband components and their working principle. Waist belt (**a**) and thigh part (**b**). Schematic showing the direction of the Exoband force and torque applied to the wearer (**c**)

A hip Exoband (Fig. 1) was fabricated. The Exoband is extremely lightweight since it is composed of textile and 3D printed parts. It is made of three main components: a waist belt (Fig. 1a) and two thigh parts, one for each limb (Fig. 1b). Each thigh part is connected to the waist belt by means of an elastic element of which the length is set by means of a ratchet strap placed in series with it. The donning time of the Exoband is under 5 min and, after learning the correct wearing guidelines, users can wear it without external help. The total weight of the Exoband is 645 g. When the thigh flexes the elastic element stretches, thus storing mechanical energy. When the leg starts to accelerate forward the elastic element initiates to shorten and applies a force in parallel with the hip flexors, ultimately assisting the user’s gait and assisting with forward acceleration of the leg (Fig. 1c). Varying the amount of preload (by means of changing the length of the ratchet strap), it is then possible to change the amount of the force applied to the wearer. Very little drift was reported by the textile components during 15 min of walking and the Exoband was rated comfortable to wear by the participants; more information on these procedures are presented in Additional file 1.

### Participants

Nine older adults were recruited (7 male, 2 female; age: 62.1 ± 5.6 yr; height: 1.71 ± 0.05 m; weight: 76.3 ± 11.9 kg, mean ± SD). They reported an absence of ongoing systemic diseases and no acute musculoskeletal injuries; in particular exclusion criteria included acute and chronic low back pain, cardiovascular or respiratory diseases, and acute lower limb musculoskeletal diseases. The study was approved by the Ethical Committee of the Department of Biomedical Sciences of the University of Padova. All participants provided written informed consent prior to testing.

### Testing protocol

**Fig. 2:**
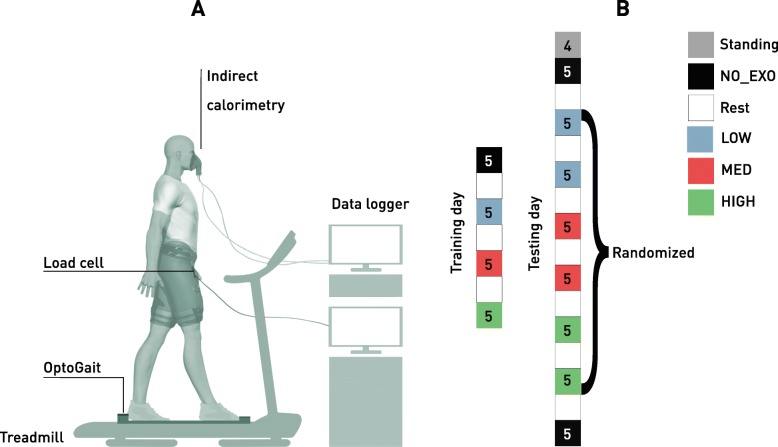
Experimental setup (**a**) and testing protocol (**b**). The numbers in the boxes represent the duration (in minutes) of each phase

The protocol was split into a training day and a testing day, with a minimum of 3 days between sessions. The aim of the training session was to familiarise participants with the assistive device and the experimental setup. Four different walking conditions were evaluated: three wearing the Exoband, with different levels of forces exerted (LOW, MED and HIGH), and one without (NO_EXO). During these conditions the participants walked on a treadmill (Technogym, Italy) at a constant speed of 1.1 m/s, since this speed is in the range of preferred walking speeds reported in older adults [27, 28]. On the training day, after a familiarisation on the treadmill, participants walked for four bouts of 5 min, experiencing each of the four testing conditions. On the testing day, a 4-min standing trial was performed at the beginning of the walking conditions to collect baseline metabolic power. After a walking warmup, participants underwent eight 5-min bouts of walking, repeating each testing condition twice. NO_EXO was performed at the beginning and at the end of the walking protocol. Following the first NO_EXO trial, two 5-min bouts for each Exoband condition (LOW, MED and HIGH) were performed in a randomised order (Fig. 2). After each walking condition, participants were given the possibility to take an adequate time of rest. To monitor relative intensities, each participant wore a heart-rate band (Polar, Finland) and also asked to indicate a number on the Borg scale [29] indicative of the perceived intensity of the exercise after 1 min and half into each bout of walking.

### Exoband setup

The force applied by the Exoband to the participants was set at the beginning of each condition (LOW, MED and HIGH) adjusting the preload of the elastic element (Fig. 1), following the procedure below.

The participants were asked to stand in a double support position, holding both feet flat on the ground. For each participant, the distance between the feet was set to match the step length, obtained from the first NO_EXO run, both for training and testing session. Holding this configuration, the elastic element of the posterior leg was then adjusted to reach a desired force level. The force applied by the Exoband was collected by means of a load cell (ISB S-Beam Force Sensor, Flintec, US) and a customized software was used to acquire force data (Labview, National Instrument, US). In each condition (LOW, MED and HIGH) the force applied by the Exoband (*Force_Exo*) was set proportionally to each participant’s mass following this equation:

$$ Force\_ Exo= ind\ast m $$

Where *ind* represents a specific force index (0.3 N/kg, 0.5 N/kg and 0.7 N/kg for LOW, MED and HIGH; respectively) and *m* the body mass of the specific participant. The same procedure was then repeated for the other leg. After this procedure, participants were asked to maintain a standing position and the preload of the elastic element was checked to ensure consistency between the two legs.

### Data collection and analysis

The force exerted on each leg by the Exoband was measured using a load cell (ISB S-Beam Force Sensor, Flintec, US), mounted between the belt and the elastic components (Fig. 2). Spatio-temporal parameters, including stance phase, swing phase, step length and stride length, were recorded using the OptoJump system (Microgate, Italy) placing its two optical bars on each side of the treadmill. Load cells and optical measurement system were synchronised by means of an external trigger box sending a 5 V square pulse. Only the last two-minutes of each 5-min bout were recorded and analysed. The average between the two bouts of each of the four conditions (LOW, MED, HIGH and NO_EXO) was used for analysis. Moreover, the first ten and the last ten force peaks for each condition of walking were extracted and compared to evaluate the consistency of force applied by the Exoband during the whole walking bouts. Force data relative to the right leg were segmented and normalised to 0–100% of the gait cycle using the heel-strike detected by the OptoJump system. Hip moment arm (*ma*) was measured on five participants and the average value (0.08 ± 0.01 m) was used to obtain an estimate of the torque applied by the Exoband to the wearer. Metabolic cost was assessed by indirect calorimetry using a gas analysis machine (Vmax, SensorMedics, US). Carbon dioxide and oxygen rate were averaged across the last 2 min (from minute 3 to 5) of each walking condition and then used to calculate metabolic rate using the Brockway equation [30]. Net metabolic rate of the four testing conditions was obtained by subtracting the standing metabolic power from the walking metabolic power of each condition and then normalising it by the body mass of each participant. The average heart rate (beats per minute, bpm), calculated across each bout of walking, was used for the statistical analysis described below.

### Statistical analysis

Statistical analysis was conducted in GraphPad Prism version 4.00 for Windows (GraphPad Software, San Diego California USA). A two-tailed paired Student’s t-test was used to assess difference in metabolic power between the NO_EXO and the Exoband condition where the participants displayed the lowest metabolic power value. One-way repeated measures analyses of variance (ANOVA) with four modes (LOW, MED, HIGH and NO_EXO) were used to assess differences between: spatio-temporal parameters (stance phase, swing phase, step length and stride length), heart rate and Borg scale. The significance level was set at *p* < 0.05 for all analyses.

## Results

### Metabolic cost

**Fig. 3:**
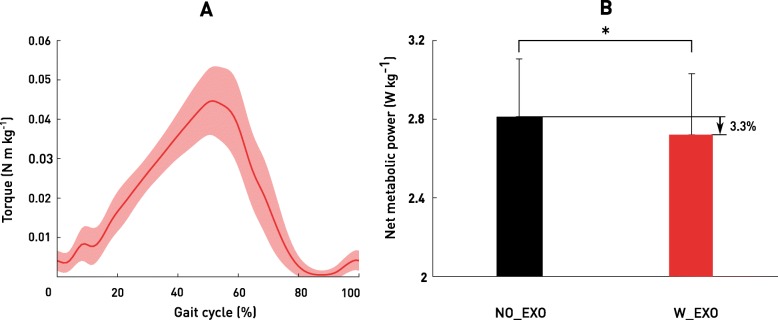
Average torque profile applied by the Exoband during the gait cycle (**a**). Net metabolic power during NO_EXO (black) and during the Exoband condition with the lowest metabolic power (red) (**b**). Data are group means ± SD. * indicates a significant difference (*p* < 0.05) with respect to NO_EXO

**Fig. 4:**
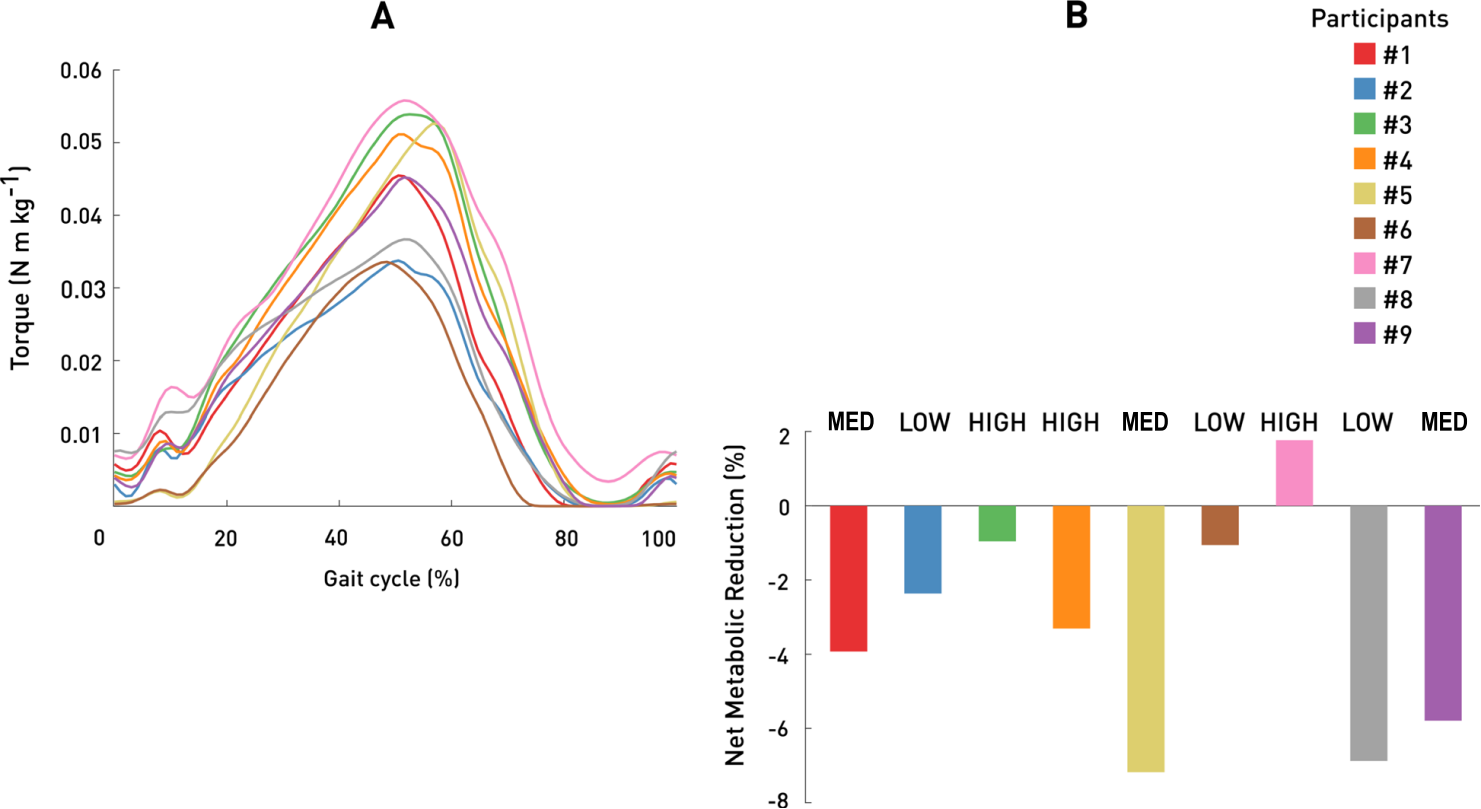
Torque applied by the Exoband during the gait cycle at which the lowest metabolic power was achieved for each participant (**a**). Highest metabolic power reduction achieved by each participant while walking with the Exoband (**b**)

Metabolic power during standing had an average value of 1.4 ± 0.2 W/kg. Eight of the nine participants involved in the study reported a reduced net metabolic power while wearing the Exoband in comparison to free walking. Specifically, six participants exhibited a reduction in metabolic power in the LOW condition, four participants in the MED condition and three in the HIGH condition with respect to free walking (Table 1). Considering the highest reduction in metabolic power reported by each participant while wearing the Exoband, this resulted in an average reduction of − 3.3 ± 3.0% (*p* = 0.02) with respect to free walking (Fig. 3b). Each participant’s highest net metabolic power reduction relative to free walking is displayed in Fig. 4b, individual net metabolic power for each condition of testing is presented in Table 1.

**Table 1:** Metabolic power for each participant during the four conditions of testing

Participant	NO_EXO [W/kg]	LOW [W/kg]	MED [W/kg]	HIGH [W/kg]
#1	2.35	2.55	2.26	2.32
#2	2.52	2.46	2.66	2.67
#3	2.96	3.13	3.28	2.93
#4	2.73	2.65	2.82	2.64
#5	3.26	3.15	3.02	3.10
#6	2.91	2.88	2.94	3.09
#7	3.16	3.39	3.41	3.22
#8	2.81	2.62	2.77	2.86
#9	2.60	2.54	2.45	2.65

### Torque assistance

**Fig. 5:**
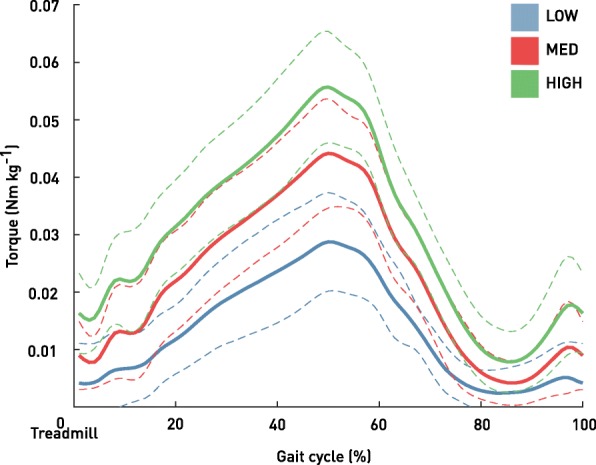
Torque profiles applied by the Exoband during the gait cycle during LOW (blue), MED (red) and HIGH (green) condition. Data are group means ± SD

Peak torques were consistent during the walking trials, with a negligible average difference between early peaks and late peaks < 0.0004 Nm/kg. The average torque profiles obtained during LOW, MED and HIGH conditions across all the participants are displayed in Fig. 5. Onset of torque profile was 11.3 ± 9.4%, 5.9 ± 5.3%, 1.6 ± 1.7% and peak torque was at 52.2 ± 2.5%, 52.2 ± 2.7% and 52.0 ± 2.4% for LOW, MED and HIGH, respectively. Each participants’ optimal torque assistive profile is displayed in Fig. 3a.

### Spatio-temporal parameters

Walking with the Exoband in the three conditions of testing did not alter the spatio-temporal parameters assessed (stance and swing phase, step and stride length) with respect to NO_EXO as shown in Table 2. Further, when we evaluated the spatio-temporal parameters in the conditions where participants exhibited their highest metabolic reduction we did not find any statistically significant difference with respect to free walking. In regard to relative intensities, there were no statistically significant differences between the four testing conditions in ratings of perceived exertion (0.69 ± 0.99, 0.97 ± 1.20, 1.03 ± 1.36, and 1.14 ± 1.57 for NO_EXO, LOW, MED and HIGH; respectively) or heart rate (92.6 ± 11.2 bpm, 92.1 ± 10.0 bpm, 93.4 ± 11.0 bpm and 92.2 ± 11.5 bpm for NO_EXO, LOW, MED and HIGH; respectively).

**Table 2:** Spatio-temporal parameters for the four conditions of testing

	NO_EXO	LOW	MED	HIGH	
Stance phase [%]	66.6 ± 1.9	66.7 ± 1.9	66.6 ± 2.1	66.5 ± 2.1	
Swing phase [%]	33.4 ± 1.9	33.3 ± 1.9	33.4 ± 2.1	33.5 ± 2.1	
Step length [m]	0.64 ± 0.03	0.64 ± 0.03	0.64 ± 0.03	0.64 ± 0.02	
Stride length [m]	1.27 ± 0.06	1.27 ± 0.05	1.28 ± 0.06	1.27 ± 0.04	

Data are presented as means ±SD

## Discussion

The aim of this study was to prove that it is possible to reduce metabolic cost in older adults by means of a simple device that assists hip flexion. Our results confirmed the potential of the Exoband as a passive system to accomplish this task. As hypothesized, walking with the Exoband had a different impact in terms of metabolic power on each participant. In particular, results indicate that the LOW condition was able to reduce the energy cost in 6 out of 9 participants (the majority of the users involved in this study), and that the highest amount of metabolic power reduction achieved by the participants who benefit from walking with the Exoband was obtained in the MED condition. Since the Exoband is a passive system, work performed by the user’s hip extensors is needed to stretch the elastic element and to store mechanical energy in it. Thus, to provide a higher assistive torque, this probably comes with a higher metabolic burden on the hip extensors which might have reduced the overall efficacy of the Exoband when applying higher torque profiles. Additionally, increasing the preload of the elastic element to achieve higher torques also had the effect of anticipating the onset of torque profile (Fig. 5). During the testing protocol we decided to change the preload of the elastic element, although this also had an impact on the onset of torque profile, for practical reasons related to the design of the Exoband itself. In the initial phase of standing (to approximately 18–20% of the gait cycle) hip extensors together with generating a flexion torque [31] were required to perform additional work to keep stretching the elastic element. This additional work performed by this muscle group might have contributed to the application of an assistive torque profile less optimal from a metabolic cost standpoint. Nevertheless, some participants displayed a reduced metabolic power also at HIGH configuration (Table 1). Although metabolic reductions were present in each of the three conditions (LOW, MED and HIGH), none of the condition per se, reported a significative reduction with respect to the NO_EXO condition, thus highlighting the need to explore individualized level of assistance and different torque profile parameters in future studies to increase the metabolic benefits provided by the Exoband.

In regard to metabolic power, the different effect observed across participants has also been reported by previous studies investigating the performance of exoskeletons for human augmentation [8, 9], and could depend on several learning [23, 24] or physiological factors [20–22]. To improve exoskeletons’ impact on metabolic power, different research groups [25, 26] applied human in-the-loop approaches to obtain an individualized level of assistance which, in turn, can lead to larger reduction of metabolic power. Although these methods are extremely valuable and hold great potential, it was not possible to apply them to the Exoband because of hardware constraints, which did not allow us to change the elastic element settings in real time during walking. Nevertheless, as an outcome of this study, this first assessment of the Exoband enabled determination of an average optimized torque (Fig. 3a) which could be used as a starting point for future exploration and further improvement of the device.

Although the magnitude of metabolic reduction reported in this study is smaller than comparative wearable devices [9, 10] an average reduction of − 3.3% can still be relevant for an older adult, since it can be equivalent to reducing the payload of ~ 3 kg [32, 33]. It is also important to highlight that our results were achieved by means of a passive device absent of actuation, electronics and battery. Other powered exoskeletons applying a torque to the hip joint have been designed by different research groups [12, 34–36] with some positive results in terms of metabolic cost reduction. To compare the metabolic cost reduction between the hip assistive devices present in the field, several methodological and developmental differences, need to be acknowledged. Further, these devices assist more than one joint [9], or both flexion and extension [36, 37] and in some cases are tethered [26, 35]; moreover, some studies performed a comparison of walking with the device powered vs walking with the device unpowered [12, 24], thus preventing a true understanding of the performance of the device in daily life tasks. A complete overview of the metabolic cost reduction reported by hip assistive devices, their type of actuation (one or more degree of freedom) and the conditions of testing investigated by the authors of these studies are presented in Table 3.

**Table 3:** Hip assistive devices and the average metabolic cost reduction they provide. The table includes information on the device type, degrees of freedom assisted and type of comparison performed by the researchers

Study	Joint assisted	Device	Comparison performed	Average metabolic reduction
Panizzolo et al. [9]	Ankle plantarflexion and hip extension	Portable and active	Powered vs No_Exo^a^	− 7.3%
Young et al. [12]	Hip extension or flexion	Portable and active	Powered vs Unpowered	−10.3% or − 9.7%
Panizzolo et al. [24]	Hip extension	Portable and active	Powered vs Unpowered	− 10.5%
Ding et al. [26]	Hip extension	Tethered and active	Powered vs No_Exo	−17.4%
Kitatani et al. [36]	Hip extension and flexion	Portable and active	Powered vs Unpowered	−10.5%
Lee et al. [37]	Hip extension and flexion	Portable and active	Powered vs No_Exo	−21.0%

^a^ No_Exo condition performed removing the effective mass of the device from the carried load

No differences were reported in spatio-temporal parameters, thus highlighting that the Exoband did not alter these gait variables. Although the invariance of spatio-temporal parameters can be an indicator of a preserved walking comfort in the use of an assistive device, at this stage, it does not give further insights into the determinants of metabolic cost reduction. We might hypothesize that this is due to reduction of hip flexors recruitment but at this stage this theory remains speculation. Further studies investigating individual joint kinematics and muscle activation are needed to explain the reported metabolic cost reduction consistent with previous reports [8, 38].

## Conclusions

The results of the present work demonstrate that a passive device composed of textile that assists the hip joint can reduce the metabolic burden of walking in older adults. Although many development challenges remain to transform this simple prototype into a viable product, this proof of concept study provides the first demonstration of a system of this kind to augment walking. This work also presented the first demonstration that a wearable device can achieve a metabolic reduction in an older adult population. Future studies will be necessary to explore the effects of different torque profiles applied by the Exoband, varying not only magnitude but also timing of assistance with the aim to maximize the effect of the elastic passive mechanism.

## Supplementary information

**Additional file 1.** Exoband drift and comfort evaluation.

## Data Availability

All data generated or analyzed during this study are included in this published article.

## Abbreviations

ANOVA: One-way repeated measures analyses of variance
HIGH: Walking condition associated with the Exoband applying a force index of 0.7 N/kg
LOW: Walking condition associated with the Exoband applying a force index of 0.3 N/kg
MED: Walking condition associated with the Exoband applying a force index of 0.5 N/kg
NO_EXO: Walking condition associated without wearing the Exoband
