# Supplementary material for: Reducing the energy cost of walking in older adults using a passive hip flexion device
Source: J Neuroeng Rehabil. 2019 Oct 15;16:117. doi: 10.1186/s12984-019-0599-4 (PMC6794907; doi:10.1186/s12984-019-0599-4)

Additional files

Additional file 1

Exoband drift and comfort

On a separate day, we conducted additional testing to evaluate Exoband comfort and drift. Fourteen male healthy participants (age: 22.3±2.7 yr; height: 1.80±4.5 m; weight: 78.5±10.0 kg, mean ± SD) were involved in this testing. Participants walked on a treadmill at a speed of 1.25 m/s wearing the Exoband with different levels of force applied (~0.15-0.35 N/kg of peak force) for 15 minutes. We placed four round yellow markers (8 mm of diameter) on anatomical landpoints of the right limb and five round yellow markers on the Exoband. The markers on the anatomical landpoints were on: knee medial epicondylus (ME), knee lateral epicondylus (LE), great trochanter (GT) and 7th cervical vertebra (C7). The markers on the Exoband were: on the thigh part on the line that connects GT to LE (A), on the thigh part on the line perpendicular to the waist belt and passing through ME (B), on the waist belt on the line that connects GT to LE (C), on the waist belt on the line perpendicular to the waist belt and passing through ME (D), in the middle of the posterior superior iliac spine (PSIS) on the waist belt (E). The distances between these points were collected at the beginning and at the end of the walking trial to assess drift of the waist belt on the different sides (LE-C, ME-D, C7-E) and of the thigh part (LE-A, ME-B) by means of a tape measurement. At the end of the walking trial, participants were asked to assess their level of comfort while wearing the Exoband on a visual analogue scale (score from 0 to 10, where 0 represents the lowest and 10 the highest level of comfort, respectively).

Results

Small drift was reported for a 15-minutes walking trial. Measurements collected indicated a shift of the waist belt of 0.34±0.36 cm (C7-E), 0.31±0.26 cm (ME-D) and 0.61±0.61 cm (LE-C). Also the thigh part drift was very small, with a shift of 0.54±0.33 cm (ME-B) and 0.45±0.40 cm (LE-A).

The Exoband was perceived comfortable by the participants, the score reported in the visual analogue scale was 7.4±1.7.

Fig. S1. Anatomical landpoints and yellow markers placed on the Exoband used for the drift analysis.

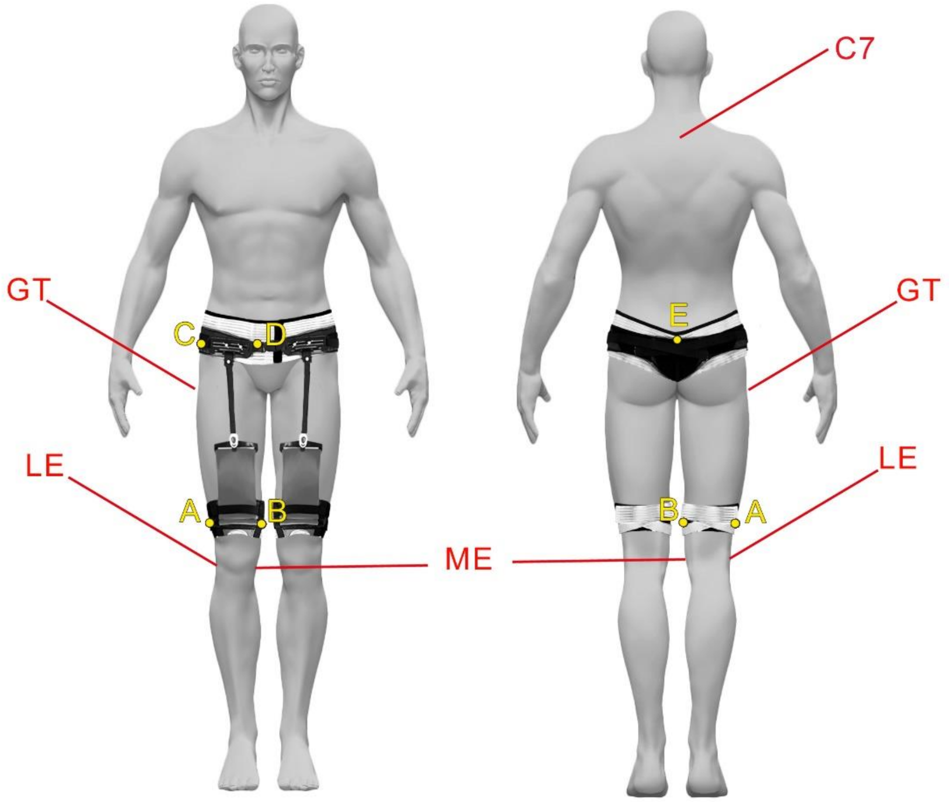

Supplement: Supplementary file 1 — Additional file 1. Exoband drift and comfort evaluation. [file 12984_2019_599_MOESM1_ESM.pdf]
